# Using a statistical learning approach to identify sociodemographic and clinical predictors of response to clozapine

**DOI:** 10.1177/02698811221078746

**Published:** 2022-02-25

**Authors:** Daniela Fonseca de Freitas, Giouliana Kadra-Scalzo, Deborah Agbedjro, Emma Francis, Isobel Ridler, Megan Pritchard, Hitesh Shetty, Aviv Segev, Cecilia Casetta, Sophie E Smart, Johnny Downs, Søren Rahn Christensen, Nikolaj Bak, Bruce J Kinon, Daniel Stahl, James H MacCabe, Richard D Hayes

**Affiliations:** 1 Institute of Psychiatry, Psychology and Neuroscience, King’s College London, London, UK; 2 South London and Maudsley NHS Foundation Trust, London, UK; 3 Shalvata Mental Health Center, Hod Hasharon, Israel; 4 Sackler Faculty of Medicine, Tel Aviv University, Tel Aviv, Israel; 5 Department of Health Sciences, Università degli Studi di Milano, Milan, Italy; 6 MRC Centre for Neuropsychiatric Genetics & Genomics, Cardiff University, Cardiff, UK; 7 H. Lundbeck A/S, Copenhagen, Denmark; 8 Cyclerion Therapeutics, Massachusetts, USA

**Keywords:** Refractory psychosis, health records, machine learning, zaponex, clorazil

## Abstract

**Background:**

A proportion of people with treatment-resistant schizophrenia fail to show improvement on clozapine treatment. Knowledge of the sociodemographic and clinical factors predicting clozapine response may be useful in developing personalised approaches to treatment.

**Methods:**

This retrospective cohort study used data from the electronic health records of the South London and Maudsley (SLaM) hospital between 2007 and 2011. Using the Least Absolute Shrinkage and Selection Operator (LASSO) regression statistical learning approach, we examined 35 sociodemographic and clinical factors’ predictive ability of response to clozapine at 3 months of treatment. Response was assessed by the level of change in the severity of the symptoms using the Clinical Global Impression (CGI) scale.

**Results:**

We identified 242 service-users with a treatment-resistant psychotic disorder who had their first trial of clozapine and continued the treatment for at least 3 months. The LASSO regression identified three predictors of response to clozapine: higher severity of illness at baseline, female gender and having a comorbid mood disorder. These factors are estimated to explain 18% of the variance in clozapine response. The model’s optimism-corrected calibration slope was 1.37, suggesting that the model will underfit when applied to new data.

**Conclusions:**

These findings suggest that women, people with a comorbid mood disorder and those who are most ill at baseline respond better to clozapine. However, the accuracy of the internally validated and recalibrated model was low. Therefore, future research should indicate whether a prediction model developed by including routinely collected data, in combination with biological information, presents adequate predictive ability to be applied in clinical settings.

## Introduction

It is estimated that up to a third of mental healthcare service-users with schizophrenia do not show an adequate response to conventional antipsychotics, or drugs for psychosis (Lally et al., 2016; Siskind et al., 2021). Clozapine is the only drug for psychosis recommended by existing guidelines for the management of treatment-resistant schizophrenia (TRS) (Lally and Gaughran, 2019; NICE, 2015; Siskind et al., 2016), with evidence showing it is associated with greater symptomatic improvement (Siskind et al., 2016) and a reduction in hospitalisation when compared to other antipsychotics (Kesserwani et al., 2019; Land et al., 2017). An episode of relapse and hospitalisation is estimated to cost over £25,000 (Munro et al., 2011), and considering the quality-adjusted life-years gained, clozapine is also the most cost-effective medication for TRS (Jin et al., 2020). However, clozapine initiation is on average delayed by 4 years (Howes et al., 2012), and there is increasing evidence that such delays are associated with a poorer response (Shah et al., 2020; Yoshimura et al., 2017). Despite its better efficacy, a recent meta-analysis estimated that, after 3 months of treatment with clozapine, between 63% and 71% of people will not show an adequate response (Siskind et al., 2017). An adequate response to clozapine is commonly defined as a reduction in symptoms of over 20% (Siskind et al., 2017), with an absolute severity below mild (Howes et al., 2017; Lieberman et al., 1994). Failure to show an adequate response to clozapine has been termed ultra-treatment resistance or clozapine-resistance (Howes et al., 2017; Lieberman et al., 1994; Shah et al., 2020).

Establishing predictors of response to clozapine could have important clinical implications for identifying clozapine-resistance earlier and initiating the augmentation of another antipsychotic or considering cessation if the expected benefits do not outweigh the side effects (Lally and Gaughran, 2019). Moreover, identifying the sociodemographic and clinical profile of people at high risk of inadequate response to clozapine may help the selection of a subgroup of patients who could be the target for pharma-led novel compound development (Vickers et al., 2006).

In a recent meta-analysis, younger age at clozapine initiation, paranoid subtype of schizophrenia and fewer negative symptoms at baseline were associated with better response (Okhuijsen-Pfeifer et al., 2020). Furthermore, another recent systematic review identified that longer duration of illness, fewer hospitalisations and fewer antipsychotic trials before clozapine initiation were associated with a better response (Griffiths et al., 2021). However, a key problem with the meta-analysis of risk factors is that individual studies adjust for different covariates making it difficult to address confounding (Griffiths et al., 2021; Okhuijsen-Pfeifer et al., 2020). Moreover, the consideration of clinical factors has been limited to examining the features of psychosis (e.g. psychosis subtype or length of illness), and as a result, comorbidities have received less attention.

Capitalising on the richness of information present in the clinical health records of the South London and Maudsley (SLaM) NHS Foundation Trust, this study aims to identify the sociodemographic and clinical predictors of response to clozapine at 3 months of treatment. To analyse an extensive range of sociodemographic and clinical factors, we used statistical learning approaches, which perform better than traditional regression analysis when the objective is to maximise predictive power, as opposed to investigating aetiological relationships (Hastie et al., 2009; Tibshirani, 1996).

## Methods

### Setting

This retrospective cohort study used data from SLaM electronic health records (EHRs). SLaM is one of the largest secondary mental healthcare providers in Europe (Stewart et al., 2009). The catchment area of this NHS Trust includes four London boroughs: Southwark, Lewisham, Lambeth and Croydon, with a population of over 1.3 million. Access to data was possible via the Clinical Record Interactive Search (CRIS) (Perera et al., 2016; Stewart et al., 2009). CRIS was established in 2007–2008, following the full implementation of EHRs in SLaM in 2006. At the time of data extraction, CRIS provided access to the de-identified information (in both EHRs structured and free-text fields) of over 230,000 individuals (Legge et al., 2016). CRIS has been approved for secondary data analysis by the Oxford C Research Ethics Committee (18/SC/0372) (Perera et al., 2016; Stewart et al., 2009).

The retrieval of information in the free-text fields is facilitated by a range of Natural Language Processing (NLP) algorithms (CRIS NLP Service, 2021; Jackson et al., 2017; Perera et al., 2016; Stewart et al., 2009). These NLP algorithms distinguish positive versus negative references to a concept of interest located within the medical record, thus outperforming a simple keyword search (Jackson et al., 2017; Perera et al., 2016). Researchers can also retrieve anonymised excerpts of free text from medical records. These can be used to validate and/or confirm results from NLP and to derive scores for validated instruments, such as the Clinical Global Impression (CGI) scale.

### Sample inclusion criteria

SLaM service-users who met the following inclusion criteria (1) had a primary diagnosis of a schizophrenia spectrum disorder (International Classification of Diseases, 10th Revision (ICD-10): F20–F29), (2) were aged between 18 and 65 years, (3) had their first trial of clozapine between 1 January 2007 and 31 December 2011 and (4) were still taking clozapine after 3 months of treatment. This cohort has been used in previous studies focusing on reasons for clozapine discontinuation (Legge et al., 2016) and antipsychotic polypharmacy before clozapine initiation (Thompson et al., 2016). For further information on sample derivation, please see Legge and colleagues’ study (Legge et al., 2016).

### Outcome measures

Response to treatment was manually rated using the CGI scale, which is a validated clinical tool to assess the patients’ overall severity of disease (CGI; Busner and Targum, 2007). Three months of treatment is the recommended length to assess the efficacy of a clozapine trial (Howes et al., 2017; Lieberman et al., 1994). Service-users’ clinical condition was retrospectively rated using information from a variety of EHRs (such as ward round notes, outpatient intervention teams notes and correspondence), which was entered around the time of clozapine initiation and 3 months later. The assessment was completed by four researchers, with an observed inter-rater reliability of .71 for the severity subscale (Thompson et al., 2016). The CGI–*severity scale* (CGI-S) ratings considered (1) the presence of psychotic symptoms, (2) the presence of negative symptoms, (3) the frequency of their occurrence, (4) the intensity or severity of symptoms and (5) the effect of symptoms on functioning in major areas of the service-users’ life (work, study, home and relationships). Severity ratings range from 1 (normal, not all ill) to 7 (among the most extremely ill patients). We calculated *change in severity* by subtracting the CGI-S ratings at baseline from those at 3 months; thus, higher values represented less improvement. It is estimated that a 1-point reduction in the CGI-S is equivalent to a 10-point reduction in Brief Psychiatric Rating Scale (BPRS) and 15-point reduction in the Positive and Negative Syndrome Scale (PANSS) scores (Leucht et al., 2006); this is clinically considered a minimal improvement

We primarily assessed response by a change in the score of severity of symptoms, as it is most commonly done (Griffiths et al., 2021; Lieberman et al., 1994); however, the response was also rated using the CGI Improvement subscale. The findings regarding the CGI Improvement scale are presented in the supplementary material (Shah et al., 2020).

### Exposure variables

Sociodemographic and clinical potential predictors of response to clozapine were measured as close as possible to the time of clozapine initiation, within the 6 months before. Sociodemographic information included gender, age at clozapine initiation, ethnicity and deprivation. Ethnicity was grouped into White (British, Irish and other White Backgrounds), Black (African, Caribbean, White and Black African, White and Black Caribbean, and any Other Black background) and Other (Bangladeshi, Chinese, Indian, Pakistani, White and Asian, any Other Asian background, any Other Mixed Background, any Other ethnic group or ethnicity not stated). The neighbourhood deprivation score of the area where the person was living at the time of clozapine initiation was based on the English Indices of Deprivation 2010 (Department for Communities and Local Government, 2011).

Clinical exposures included the ICD-10 psychiatric diagnoses present in the health records within the 6 months before clozapine initiation, the clinical condition assessment using the Health of the Nation Outcome Scale (HoNOS) and the length of illness. Psychiatric diagnosis was identified from information in the EHR structured fields and information in free-text fields using NLP applications designed for that purpose (CRIS NLP Service, 2021). Where more than one diagnosis was recorded, a diagnostic hierarchy was used: in patients with diagnoses of both schizoaffective disorder (F25) and schizophrenia (F20), schizoaffective disorder was taken as the diagnosis. The other codes for psychotic disorders (F21–F24, F28–F29) were only used in patients with no instances of F20 or F25. Psychiatric comorbidities mentioned in records within 6 months before clozapine initiation were also included in the analyses. These comprised personality disorders (F60–F61); any substance use disorders (F10–F14, F16, F18–F19); developmental disorders (F70–F79, F80–F84, F88, F90); anxiety-related disorders (F40–F43); and mood disorders (F30–F39, F42.1). The HoNOS (Pirkis et al., 2005; Wing et al., 1998) was considered as evidence of service-users’ psychiatric symptoms, problematic behaviour, level of impairment and problems in social functioning. The HoNOS is completed regularly as part of routine clinical care in SLaM. The 12 items of the scale are rated between 0 (no problem), 1 (minor problem requiring no action), 2 (mild problem but definitely present), 3 (moderately severe problem) and 4 (severe to very severe problem). Due to low numbers in some of the categories, we collapsed the score into 0 (no problem), 1 (minor problem requiring no action) and 2–4 (mild to very severe problem) (Hayes et al., 2012). The most relevant HoNOS score was selected using a hierarchy in reference to the date of clozapine initiation: the closest date to initiation within the 3 months before; if none available, up to 1 week after; if also unavailable, the latest date between 3 months and 1 year before clozapine initiation; if also unavailable, HoNOS was coded as missing. The length of psychotic illness at the time of clozapine initiation was calculated based on the information in case notes. Depending on the information available, it could refer to first psychotic symptoms, prescription of a drug for psychosis, diagnosis of psychosis or the first contact with services.

Several measures of service use in the 6 months before clozapine initiation were included in the models, namely: the number of days as an inpatient, the number of days where there was at least one face-to-face contact with outpatient intervention teams (maximum one contact per day) and the number of outpatient intervention team events (not restricted to one per day and including events where there was non-contact with the patient). To adjust for differences in the period receiving care in SLaM, these measures were divided by the number of days that the person was under active treatment with SLaM (active days) during the 6 months before clozapine initiation. Furthermore, for the regression models, due to skewed values, these ratios were log-transformed. Other measures of service use, in the 6 months before clozapine initiation, included having received care from an early intervention service for psychosis, having received care from a psychiatric intensive care unit, the number of compulsory medical hospitalisations under the Mental Health Act 1983 (MHA) (HM Government, 1983), having been detained under a forensic section of the MHA and having been conveyed to a place of safety by police from a public place or private premises, all measured in the 6 months before clozapine initiation.

To assess possible non-adherence to treatment in the 6 months before the clozapine trial, we analysed evidence of treatment with a Long-Acting Injection (LAI; depot) drug for psychosis or having been submitted to supervised pharmacological treatment in the community, a Community Treatment Order (CTO) under the MHA (Barnes et al., 2020; Kadra et al., 2016; MH Government, 2007).

### Statistical analysis

We examined predictors of clozapine non-response using a linear Least Absolute Shrinkage and Selection Operator (LASSO) regression (Tibshirani, 1996). We chose regularised regression over traditional statistical methods to minimise the variance of prediction and overfitting and to perform automatic variable selection (Hastie et al., 2009). We followed the standard guidelines for model building available at the time of study initiation, including the steps proposed by Steyerberg and Vergouwe (2014). We reported the results according to the TRIPOD statement (Collins et al., 2015). The missing data were imputed through K-nearest neighbours (Jonsson and Claes, 2004), using the Gower distance (Gower, 1971), and LASSO regression was fitted with 20-time repeated 10-fold cross-validation tuning on a grid of 100 tuning parameters, minimising the mean squared error (MSE). The model’s discriminative performance was evaluated with a pseudo-*R*^2^ defined as 1−MSE/var(y) , with y being the outcome (Breiman, 2001). Calibration, a measure of the agreement between observed values and predictions, was assessed with the calibration slope and the calibration-in-the-large. The calibration slope regards the slope of the line with the best fit obtained by regressing the observed outcome on the predicted outcome; the calibration-in-the-large is the difference between the mean of the observed outcome and the mean of the predicted outcome (Steyerberg, 2019). All measures of performance were internally validated using 100-time repeated 10-fold cross-validation optimism-correction after the method of Harrell (2015). When the calibration slope departed from the ideal slope of 1, the estimated coefficients were recalibrated by multiplying them by the corrected calibration slope; the intercept of the model was recalibrated by multiplying it by the corrected calibration slope and by adding the corrected calibration-in-the-large to the result (Steyerberg, 2019). Calibration plots of observed outcomes against predictions (uncalibrated line) and against recalibrated predictions (recalibrated line) are presented. Analyses were undertaken in R software using the following packages: glmnet (Friedman et al., 2010), caret (Kuhn, 2008), pROC (Robin et al., 2019), StatMatch (D’Orazio, 2015) and c060 (Sill et al., 2014).

## Results

### Participants

There were 316 service-users with a schizophrenia spectrum disorder who had their first trial of clozapine between 2007 and 2011 (Legge et al., 2016). Of these, 242 (76.6%) were treated for longer than 3 months and were considered eligible for this study. Men comprised 67% of the cohort, and people with minority ethnic background 59%. At the time of clozapine initiation, the median age of the sample was 35.9 years, and the median length of psychotic illness was 8 years. Eighty-seven per cent were diagnosed with schizophrenia (F20), and 24.4% had a comorbid mood disorder (F30–F39). At the time of clozapine initiation, 51% were hospitalised (and 30% of the whole sample was hospitalised involuntarily under the MHA). Sixty-eight per cent had received a depot medication in the 6 months before clozapine initiation. Missing data on at least one variable were present in 58 (28%) cases. Table 1 shows the sample characteristics, clinical factors, service use and missing data.

**Table 1.** Descriptive statistics for the exposures included in the models.

Exposures	*N* = 242	Missing data
Continuous	Median (25th–75th p)	Count (%)
Categorical	Count (%)	
*Sociodemographic information*		
Age (years)	35.9 (28.2–44.0)	0
Gender		
Men (R)	162 (66.9)	
Women	80 (33.1)	0
Ethnicity		
Black (R)	118 (48.8)	0
White	100 (41.3)	
Other	24 (9.9)	
Neighbourhood deprivation score	31.2 (23.9–37.0)	13 (5.4)
*Main diagnosis*		
Schizophrenia spectrum diagnosis		
Schizophrenia (R) (ICD-10: F20)	211 (87.2)	0
Schizoaffective (ICD-10: F25)	26 (10.7)	
Other prolonged psychosis (ICD-10: F21–F24.9, F28–F29.9)	5 (2.1)	
Length of illness (years)	8.0 (3.5–14.0)	11 (4.6)
*Comorbidities* (diagnosis in the 6 months before)		
Personality disorder (ICD-10: F60–F61)	27 (11.2)	0
Any substance use disorder (ICD-10: F10–F14, F16, F18–F19)	36 (14.9)	0
Developmental disorder (ICD-10: F70–F79, F80–F84, F88, F90)	9 (3.7)	0
Anxiety-related disorder (ICD-10: F40–F43)	8 (3.3)	0
Mood disorder (ICD-10: F30–F39, F42.1)	59 (24.4)	0
*The Health of the Nation Outcome Scales* (closest to clozapine initiation but between 1 year before and 1 week after)		
1. Overactive, agitated behaviour		
No problem (R)	93 (38.4)	29 (12.0)
Minor problem, no action	55 (22.7)	
Significant problem	65 (26.9)	
2. Non-accidental self-injury		
No problem (R)	168 (69.4)	29 (12.0)
Minor problem	24 (9.9)	
Significant problem	21 (8.7)	
3. Drinking or drug-taking		
No problem (R)	146 (60.3)	32 (13.2)
Minor problem	18 (7.4)	
Significant problem	46 (19.0)	
4. Cognitive problems		
No problem (R)	100 (41.3)	32 (13.2)
Problem	55 (22.7)	
Significant problem	55 (22.7)	
5. Physical illness or disability		
No problem (R)	151 (62.4)	29 (12.0)
Minor problem	37 (15.3)	
Significant problem	25 (10.3)	
6. Hallucinations and delusions		
No problem (R)	19 (7.9)	33 (13.6)
Minor problem	24 (9.9)	
Significant problem	166 (68.6)	
7. Depressed mood		
No problem (R)	94 (38.8)	30 (12.4)
Minor problem	65 (26.9)	
Significant problem	53 (21.9)	
8. Other mental and behavioural problems		
No problem (R)	56 (23.1)	31 (12.8)
Minor problem	41 (16.9)	
Significant problem	114 (47.1)	
9. Relationship problems		
No problem (R)	69 (23.1)	31 (12.8)
Minor problem	64 (16.9)	
Significant problem	78 (47.1)	
10. Activities of daily living		
No problem (R)	83 (34.3)	29 (12.0)
Minor problem	55 (22.7)	
Significant problem	75 (31.0)	
11. Living conditions		
No problem (R)	111 (45.9)	39 (16.1)
Minor problem	44 (18.2)	
Significant problem	48 (19.8)	
12. Occupation and activities		
No problem (R)	69 (28.5)	39 (16.1)
Minor problem	56 (23.1)	
Significant problem	78 (32.2)	
8.a Other mental and behavioural problems. Type:		
Phobic, anxiety, obsessive-compulsive	50 (20.7)	29 (12.0)
Mental strain/tension	39 (16.1)	29 (12.0)
Dissociative, somatoform	13 (5.4)	29 (12.0)
Eating, sleep, sexual	43 (17.8)	29 (12.0)
*Service use* (in 6 months before clozapine initiation)		
Days with face-to-face clinical contacts with outpatient intervention teams/active days	0.07 (0.03–0.11)	0
Days in hospitalisation/active days	0.29 (0.04–0.73)	0
Number of outpatient intervention teams events/active days	0.15 (0.08–0.27)	0
Received care from a psychiatric intensive care unit	26 (10.7)	0
Received care from an early intervention service for psychosis	33 (13.6)	0
Conveyed to a place of safety by police (MHA, police sections)	17 (7.0)	0
Detained under the forensic section of the (MHA Part 3 sections)	24 (9.9)	0
Count of compulsory medical hospitalisations (MHA Part 2 sections)	0 (0–56.8)	0
*Non-adherence* (in the 6 months before clozapine initiation)		
Supervised community treatment (Community treatment order)	3 (1.2)	0
Long-Acting Injection (depot) drug for psychosis	165 (68.2)	0
*Clinical Global Impression Scale*		
CGI severity at baseline	5.0 (5.0–6.0)	0

p: percentile; R: reference category; MHA: Mental Health Act 1983; HoNOS: Health of the Nation Outcome Scales; CGI: Clinical Global Impression; ICD-10: International Classification of Diseases, 10th Revision.

### Predictors of response to clozapine

After 3 months of clozapine initiation, 55% of the cases showed a minimal response (i.e., 1-point reduction in severity) and 22% showed no change in the severity of symptoms (Table 2). Moreover, only 18% were observed to be within the threshold of mild severity (Howes et al., 2017; Lieberman et al., 1994). According to the LASSO regression, three factors were identified as predictors of better response to clozapine: higher severity at baseline, female gender and having a comorbid mood disorder (Table 3). We checked the robustness of high severity predicting better response by examining potential ceiling, floor and regression to the mean effects. No such effects were observed.

**Table 2.** Clinical Global Impression–Severity scale scores at baseline and at 3 months of treatment with clozapine.

CGI–Severity	At baseline	At 3 months	Change in severity of symptoms	
Scores	*n* (%)	*n* (%)	Scores	*n* (%)
1 (normal)	0	0	−4 (reduction)	1 (0)
2 (borderline ill)	0	3 (1)	−3	7 (3)
3 (mildly ill)	0	42 (17)	−2	38 (20)
4 (moderately ill)	37 (15)	129 (53)	−1	133 (55)
5 (markedly ill)	129 (53)	60 (25)	0 (no change)	53 (22)
6 (severely ill)	75 (31)	8 (3)		
7 (extremely ill)	1 (0)	0		
Mdn (IQR)	5 (5, 6)	4 (4, 5)		−1 (−1, −1)

CGI: Clinical Global Impression; IQR: interquartile range.

**Table 3.** LASSO regression selected predictors for severity change at 3 months.

Severity change (*higher scores indicate poorer response: −4 to −1* = *reduction in severity; 0* = *no change*)	Mean change	Recalibrated coefficients
(Intercept)	0.695	0.9200
Baseline CGI–Severity score	−0.358	−0.4907
Female gender	−0.167	−0.2287
Comorbid mood disorder	−0.030	−0.0412
*Model performance*	Apparent	Corrected
Pseudo *R*^2^	0.21	0.18
Calibration slope	1.36	1.37
Calibration-in-the-large	0.00	−0.01

CGI: Clinical Global Impression; LASSO: Least Absolute Shrinkage and Selection Operator.

**Figure 1.**
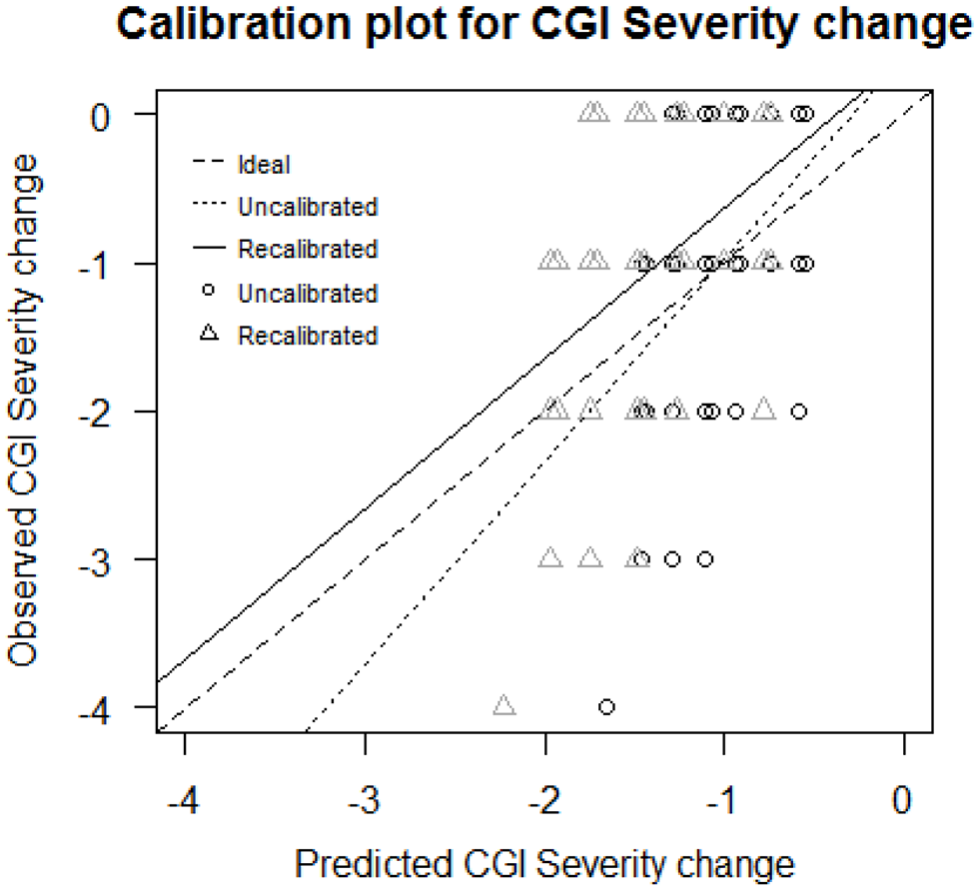
Calibration plot of the model predicting change in the severity of symptoms. For the same *y*-coordinate, the circles’ *x*-coordinate is the predicted outcome through the uncalibrated model and the triangles’ *x*-coordinate is the predicted outcome through the recalibrated model.

The internally validated pseudo-*R*^2^ was 0.18, which indicates these three factors should explain 18% of the variance in response to clozapine in unseen samples of the same clinical population. The optimism-corrected calibration slope was 1.37, indicating underfitting of the model if the model is applied to unseen data (Table 3). The calibration plot shows that the recalibrated model presents a calibration line closer to the ideal calibration line (45° line) than the calibration line of the non-calibrated model (Figure 1).

## Discussion

This study aimed to identify sociodemographic and clinical predictors of response to clozapine at 3 months of treatment. Of the 242 SLaM service-users who had a 3-month trial of clozapine, 22% showed no change in severity, while 55% showed a minimal response, and only 18% were considered to be mildly ill or better (Howes et al., 2017; Leucht et al., 2006; Lieberman et al., 1994). These findings are in line with previous observations that up to 71% of people treated with clozapine will not show an adequate response in the short term (Lieberman et al., 1994; Siskind et al., 2017).

Regarding the sociodemographic predictors of clozapine response, women appear to show a better response than men. A similar result was seen in Usall et al. (2007) study, where the outcome was also measured using the CGI, and also in Shah et al. (2020), where late non-response to clozapine was more frequent in men. However, the direction of the effect of gender differences in clozapine response is not consistent across studies (Griffiths et al., 2021; Okhuijsen-Pfeifer et al., 2020; Yoshimura et al., 2017). CGI captures a broader range of domains (e.g. daily activities) than the traditional symptom-based measures (e.g. PANSS). It may be that women are more likely to show response in these other domains, hence their superior response on the CGI.

In previous studies, both younger age and shorter duration of illness at the time of the clozapine trial have been associated with better response (Griffiths et al., 2021; Okhuijsen-Pfeifer et al., 2020). Age did not emerge as a predictor of response in our study. However, it is notable that our cohort was older than in many studies (median age 35.9), and the median time to clozapine treatment was rather long, at 8 years. There is evidence to suggest that a shorter length of illness and younger age at onset are associated with best responses only when clozapine is introduced within the first 3 years of illness (Yoshimura et al., 2017). It may be that our study’s relatively older cohort contained a large proportion of people who had missed this window, diluting the effect of length of illness and age.

Concerning other clinical factors, more severe symptoms at baseline predicted a better response to clozapine. In a recent meta-analysis of observational studies, no significant associations between global baseline scores (from studies using CGI, BPRS and PANSS, analysed separately) and response were observed; only fewer negative symptoms (using PANSS) were associated with better response (Okhuijsen-Pfeifer et al., 2020). Also, in a meta-analysis of randomised controlled trials, comparing the efficacy of clozapine versus other antipsychotics, it was observed that higher baseline mean psychosis score predicted greater response for clozapine in the long term (⩾3 months), but not in the short term (<3 months) (Siskind et al., 2016). According to the nature of the scoring of the scale, and attending to the fact that complete remission of symptoms in schizophrenia is rare, it is possible that the finding of high severity at baseline predicting better response is due to the most severe cases at baseline showing a larger reduction in symptoms after 3 months.

Having a comorbid mood disorder predicted a greater reduction in severity following treatment with clozapine. Research on the association between response to clozapine and psychiatric comorbidities is scarce. However, in one previous study, no differences in psychiatric comorbidities between long-term clozapine responders and non-responders were reported (Shah et al., 2020). Depressive disorders seem to be more prevalent in TRS samples (Jönsson et al., 2019; Wimberley et al., 2016), but, to our knowledge, this is the first time a comorbid mood disorder is associated with a better response to clozapine. This finding is in line with the evidence that clozapine has some efficacy in mood disorders, including bipolar disorder (Li et al., 2015).

The novelty of some of this study’s observed associations, or the lack thereof, warrants caution in the conclusions to be drawn. The use of statistical learning statistical methods that are focused on prediction instead of the traditional statistical methods, which are fit for analyses of existing (past) associations, may be related to the divergence in findings between this study and the previous literature. Moreover, most past research has adopted a different way to measure response, namely, for the dichotomisation of response levels (Okhuijsen-Pfeifer et al., 2020). The impact of these methodological differences is unknown, so it is imperative that future research using similar methods is conducted in order to establish a more solid knowledge.

### Limitations and strengths

This study had several limitations that need to be borne in mind when interpreting the findings. First, given that this study used secondary data, we were restricted to the information that is routinely collected in SLaM healthcare provision. Second, previous studies showed that clozapine dose, the number of previous antipsychotic trials and delays in clozapine initiation are key predictors of clozapine response, and these factors were not analysed in the present study (Nielsen et al., 2012; Shah et al., 2020; Yoshimura et al., 2017). Third, the statistical models were not tested in other samples for external validity, given the lack of data. Finally, and most importantly, our analysis may be underpowered. In contrast to what is recommended by a large part of the literature (Hastie et al., 2009; Tibshirani, 1996; Wang et al., 2020), regularised regression methods using the penalty optimising the error (the best penalty) do not always resolve problems associated with limited sample size relative to the number of variables (Van Calster et al., 2020), especially if the irrepresentable condition (where relevant variables should not strongly correlate with irrelevant variables) does not hold (Zhao and Yu, 2006). Therefore, developing LASSO prediction models using the best penalty with small samples and a relatively large number of predictors could potentially lead to overfitting of the models and poor model performance. Riley et al. (2019) suggest a sample size calculation for linear prediction models to avoid overfitting. According to their research, a model like the one we have developed, including 42 parameters, expecting to have an adjusted Cox–Snell *R*^2^ of 0.2 with our outcome mean and standard deviation, would need a minimum sample size of 1602 to avoid overfitting, which is much larger than our study sample size. It is also true that Riley et al. (2019) do not provide a sample size calculation adapted to regularised regression models (which do not retain all the variables in the model), and there is no explicit guidance about the sufficient sample size to avoid overfitting for LASSO regression. Nonetheless, in the present study, we have corrected the models’ overfitting/underfitting with internal validation and recalibration, which leads to the best predictive performance possible using LASSO with these data. We consider that the alternative of using different statistical learning methods could lower the interpretability of the results.

A key strength of this study is the representativeness of the population studied and the likely low selection bias. In the United Kingdom, almost all people with severe mental illness receive free medical care through the NHS, and SLaM is the only NHS provider of secondary care for mental health in its catchment area. Another strength of this study is investigating a vast range of sociodemographic, clinical factors and service-use events available in clinical records. Similarly, the depth and size of the information on the electronic records have enabled us to combine extensive information to inform the CGI ratings.

## Conclusions

Our findings suggest that women, people with a comorbid mood disorder and those who are most ill, according to the CGI-S, respond better to treatment with clozapine. Sociodemographic and clinical factors may have insufficient predictive power alone for the development of clinical predictive algorithms (as these factors are associated with only 18% of variance in response); however, future research could determine whether this could potentially be useful in combination with information regarding genetic factors and other biomarkers.

## Supplemental Material

sj-docx-1-jop-10.1177_02698811221078746 – Supplemental material for Using a statistical learning approach to identify sociodemographic and clinical predictors of response to clozapine Supplemental material, sj-docx-1-jop-10.1177_02698811221078746 for Using a statistical learning approach to identify sociodemographic and clinical predictors of response to clozapine by Daniela Fonseca de Freitas, Giouliana Kadra-Scalzo, Deborah Agbedjro, Emma Francis, Isobel Ridler, Megan Pritchard, Hitesh Shetty, Aviv Segev, Cecilia Casetta, Sophie E Smart, Johnny Downs, Søren Rahn Christensen, Nikolaj Bak, Bruce J Kinon, Daniel Stahl, James H MacCabe and Richard D Hayes in Journal of Psychopharmacology
