# Supplementary material for: Using a statistical learning approach to identify sociodemographic and clinical predictors of response to clozapine
Source: J Psychopharmacol. 2022 Feb 25;36(4):498–506. doi: 10.1177/02698811221078746 (PMC9066692; doi:10.1177/02698811221078746)
Supplement: sj-docx-1-jop-10.1177_02698811221078746 – Supplemental material for Using a statistical learning approach to identify sociodemographic and clinical predictors of response to clozapine [file sj-docx-1-jop-10.1177_02698811221078746.docx]

**Supplementary material**

**Table S1.** *Clinical Global Impression – Improvement subscale scores at three months of treatment with clozapine*

| ***CGI –Improvement scores*** | *n (%)* |
| --- | --- |
| 1 (very much improved) | 13 (5%) |
| 2 (much improved) | 132 (55%) |
| 3 (minimally improved) | 65 (27%) |
| 4 (no change) | 31 (13%) |
| 5 (minimally worse) | 1 (0%) |
| 6 (much worse) | 0 |
| 7 (very much worse) | 0 |
| *Mdn (IQR)* | 2 (2, 3) |

**Table S2.** *LASSO regression selected predictors for less improvement* *at three months*

| **GCI improvement ratings**  *(higher scores indicate poorer response: 1 = very much improved, 5 = minimally worse)* | Mean change | Recalibrated coefficients |
| --- | --- | --- |
| Intercept | 2.4306 | 3.1987 |
| Female gender | -0.3019 | -0.3974 |
| Supervised community treatment (CTO) | 0.1324 | 0.1743 |
| HoNOS occupational problems (minor problem) | -0.1280 | -0.1685 |
| Comorbid mood disorder | -0.1243 | -0.1636 |
| HoNOS living conditions problems (minor problem) | -0.1048 | -0.1379 |
| HoNOS relationship problems (significant problem) | 0.0254 | 0.0334 |
| HoNOS physical illness (significant problem) | 0.0186 | 0.0245 |
| Neighbourhood deprivation score | -0.0033 | -0.0043 |
| Length of illness | 0.0024 | 0.0032 |
| Received care from an early intervention service for psychosis | -0.0002 | -0.0003 |
| Number of outpatient intervention teams events/active days (log transformed) | -0.0001 | -0.0001 |
| *Model performance* | Apparent | Corrected |
| Pseudo R-squared | 0.12 | 0.08 |
| Calibration slope | 1.89 | 1.32 |
| Calibration-in-the-large | 0.00 | -0.01 |


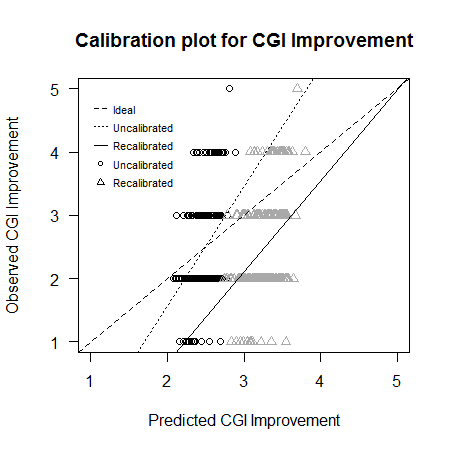


**Figure S1.** Calibration plot of the model predicting non-response using the CGI-Improvement scale. For the same y-coordinate, the circles’ x-coordinate is the predicted outcome through the uncalibrated model and the triangles’ x-coordinate is the predicted outcome through the recalibrated model.
